# Hydroxy-epoxide and keto-epoxide derivatives of linoleic acid activate trigeminal neurons

**DOI:** 10.1016/j.ynpai.2020.100046

**Published:** 2020-04-30

**Authors:** Suzanne Doolen, Gregory S. Keyes, Christopher E. Ramsden

**Affiliations:** a Department of Physiology, University of Kentucky, 800 Rose Street, Lexington, KY 40536-0298, United States; b Lipid Peroxidation Unit, Laboratory of Clinical Investigation, National Institute on Aging, National Institutes of Health (NIH), Baltimore, MD 21224, USA; c Intramural Program of the National Institute on Alcohol Abuse and Alcoholism, NIH, Bethesda, MD 20814, USA

**Keywords:** LA, linoleic acid, 11H-12,13E-LA, 11-hydroxy-12,13-*trans*-epoxy-(*9Z*)-octadecenoate, 11H-9,10E-LA, 11-hydroxy-9,10-*trans*-epoxy-(*12Z*)-octadecenoate, 11-HEL, 11-hydroxy-epoxide-linoleic acid, CGRP, calcitonin gene related peptide, 9-HODE, 9-hydroxy-octadecadienoic acid, PGE_2_, prostaglandin E_2_, TN, trigeminal neuron, HpODEs, hydroperoxy-octadecadienoic acids, HODEs, octadecadienoic acids, aCSF, artificial cerebrospinal fluid, EpOMEs, epoxy-octadecenoic, DiHOMEs, dihydroxy-octadecenoic acids, Pain, Hyperalgesia, Linoleic acid, Peroxidation, Oxylipin

## Abstract

Endogenous lipid mediators are proposed to contribute to headache and facial pain by activating trigeminal neurons (TN). We recently identified 11-hydroxy-epoxide- and 11-keto-epoxide derivatives of linoleic acid (LA) that are present in human skin and plasma and potentially contribute to nociception. Here we expand upon initial findings by examining the effects of 11-hydroxy- and 11-keto-epoxide-LA derivatives on TN activation in comparison to LA, the LA derivative [9-hydroxy-octadecadienoic acid (9-HODE)] and prostaglandin E_2_ (PGE_2_). 11-hydroxy- and 11-keto-epoxide-LA derivatives elicited Ca^2+^ transients in TN subpopulations. The proportion of neurons responding to test compounds (5 μM, 5 min) ranged from 16.2 ± 3.8 cells (11 K-9,10E-LA) to 34.1 ± 2.4 cells (11H-12,13E-LA). LA and 9-HODE (5 μM, 5 min) elicited responses in 11.6 ± 3.1% and 9.7 ± 3.4% of neurons, respectively. 11H-12,13E-LA, 11K-12,13E-LA, and 11H-9,10E-LA produced Ca^2+^ responses in significantly higher proportions of neurons compared to either LA or 9-HODE (F (6, 36) = 5.12, P = 0.0007). 11H-12,13E-LA and 11H-9,10E-LA increased proportions of responsive neurons in a concentration-dependent fashion, similar to PGE_2_. Most sensitive neurons responded to additional algesic agents (32.9% to capsaicin, 40.1% to PGE_2_, 58.0% to AITC), however 20.6% did not respond to any other agent. In summary, 11-hydroxy-epoxide derivatives of LA increase trigeminal neuron excitability, suggesting a potential role in headache or facial pain.

## Highlights

• 11-hydroxy- and 11-keto-epoxide-LA derivatives elicit Ca^2+^ transients in trigeminal neuron subpopulations.
• 11H-12,13E-LA, 11 K-12,13E-LA, and 11H-9,10E-LA produce Ca^2+^ responses in higher proportions of neurons than linoleic acid or 9-HODE.
• 11-hydroxy-epoxide- and 11-keto-epoxide derivatives of linoleic acid potentially contribute to nociception.

## 1 Introduction

Headache and facial pain syndromes are common, consequential sources of suffering, disability, and societal expense that are linked to trigeminal neuron (TN) sensitization (Iyengar et al., 2019, Shen et al., 2019, Shetty et al., 2018). The identification of new mediators and mechanisms underlying TN activation and/or sensitization could provide leads for development of new therapeutics (Ashina et al., 2019). We recently identified two 11-hydroxy-epoxide- derivatives of linoleic acid (LA)—11-hydroxy-12,13-*trans*-epoxy-(*9Z*)-octadecenoate (abbreviated 11H-12,13E-LA) and 11-hydroxy-9,10-*trans*-epoxy-(*12Z*)-octadecenoate (abbreviated 11H-9,10E-LA)—in inflamed human skin. These 11-hydroxy-epoxide-linoleic acid (11-HEL) derived oxylipins were observed to sensitize rat dorsal root ganglia neurons to release calcitonin gene related peptide (CGRP) (Ramsden et al., 2017). 11H-12,13E-LA injection evoked rodent pain-related behavior *in vivo*, and reductions in circulating 11H-12,13E-LA correlated with headache relief in a small clinical trial, further suggesting that these LA–derived mediators might contribute to sensory signaling (Ramsden et al., 2017). These 11-HELs share a 3-hydroxy-*Z*-pentenyl-*E*-epoxide moiety with the previously described bioactive hepoxilin B3 derivatives of arachidonic acid (Anton and Puig, 1998), suggesting that this substructure could mediate nociceptor sensitization.

**Fig. 1:**
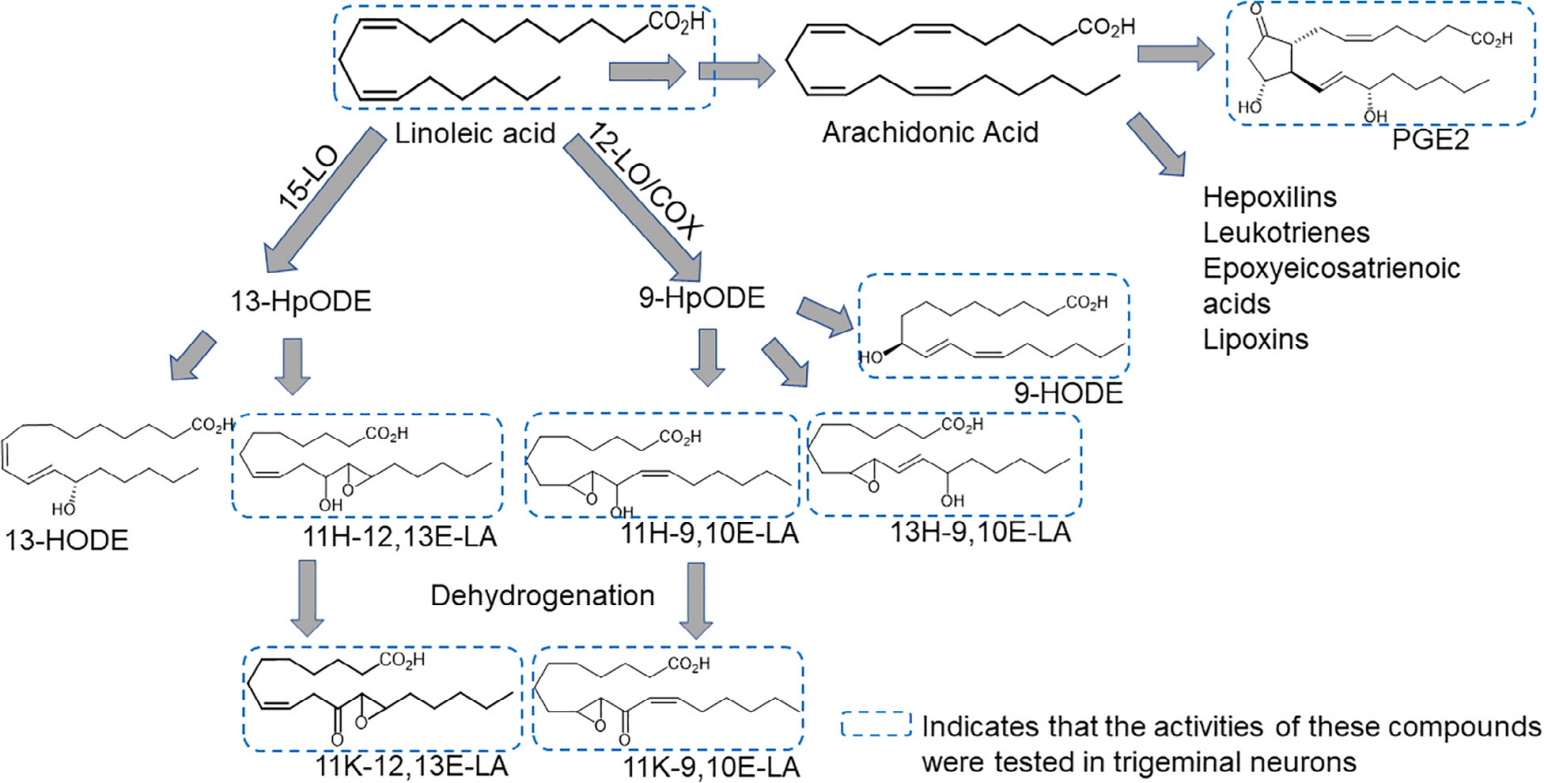
Proposed molecular pathways involved in the synthesis of compounds tested in TNs. (A) Linoleic acid undergoes enzyme or free radical mediated peroxidation to form 9- and 13-hydroperoxy-octadecadienoic acids (HpODEs), which can be further converted to hydroxy-octadecadienoic acids (HODEs) or hydroxy-epoxides including 11H-12,13E-LA and 11H-9,10E-LA. Hydroxy-epoxides can be dehydrogenated to form keto-epoxides such as 11K-12,13E-LA and 11K-9,10E-LA. Arachidonic acid is the precursor to PGE_2_ and other classic nociceptive mediators.

**Fig. 2:**
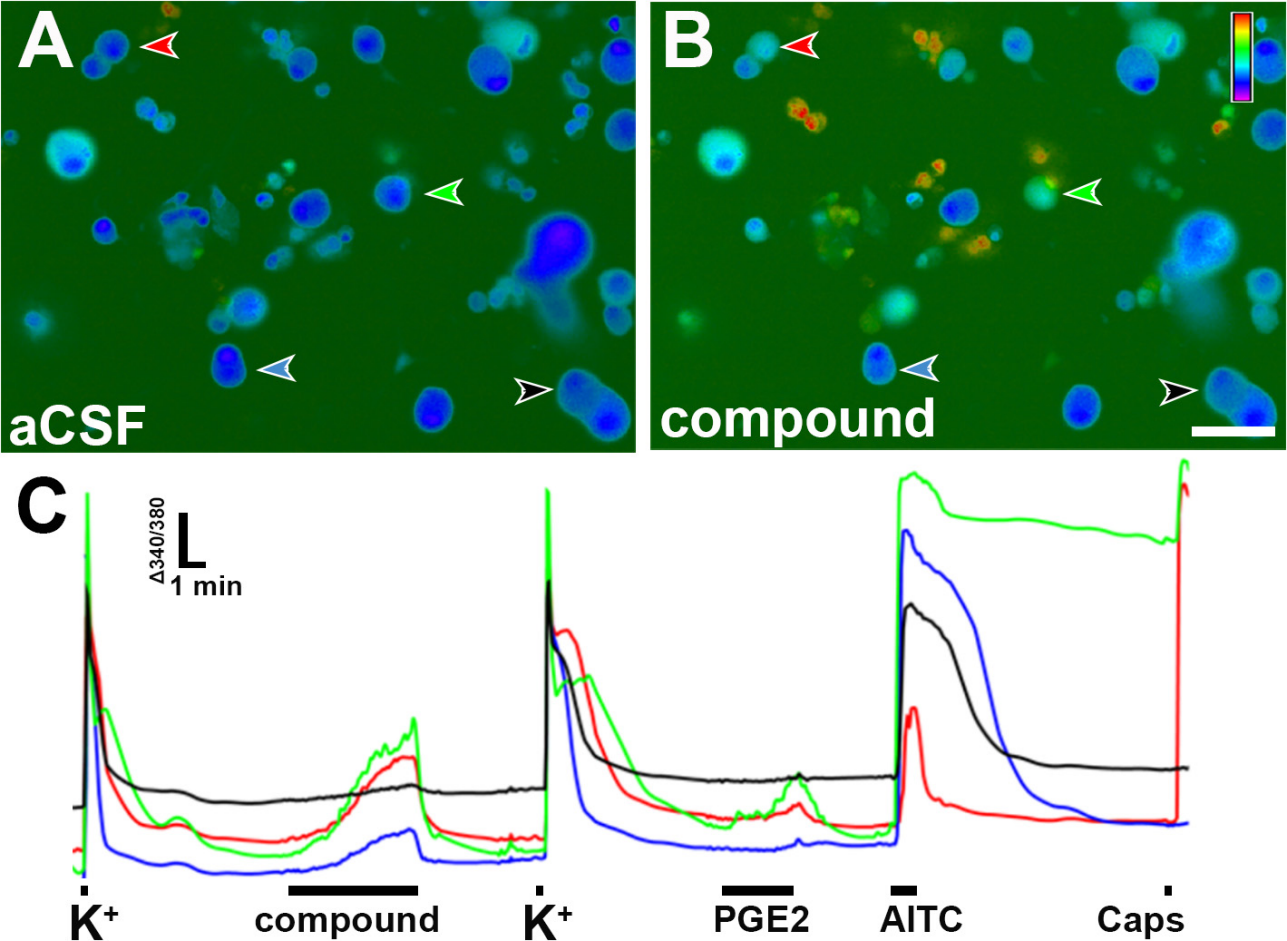
Oxylipins produce Ca^2+^ transients in TNs. Representative images of trigeminal ganglion cell Ca^2+^ signals in at rest (A; aCSF) and in the presence of tested compounds (11-hydroxy-epoxide and 11-keto-epoxide derivatives of LA) (B; oxylipin compound). (C) Change is 340/380 ratios vs. time for TNs shown in A-B. The color of arrowheads in A-B match the color of the trace in (C). Timing of each chemical application is indicated by black bars below traces.

Based on these observations, we hypothesized that 11-HELs and/or their metabolic derivatives could contribute to TN sensitization. The aims of this study were to: (1) determine the effects of these 11-HELs and their 11-keto-epoxide-linoleate (11-KEL) derivatives on activation of mouse TNs, compared to the established LA derivative [9-hydroxy-octadecadienoic acid (9-HODE)] and the classic arachidonic acid-derived nociceptive mediator prostaglandin E_2_ (PGE_2_; Fig. 1), and (2) take the first steps toward characterizing the TN subpopulations affected by each mediator.

## 2 Materials and methods

### 2.1 Animals

For TN sensitization studies, male C57Bl/6 mice purchased from Charles River Laboratories (Indianapolis, USA) were housed four to a cage and had access to food and water ad libitum. All animal procedures were conducted at the University of Kentucky and were approved by the Institutional Animal Care and Use Committee at the University of Kentucky in accordance with American Veterinary Medical Association guidelines. Mice were maintained in a temperature (68–72° F) and humidity (30–70%) controlled environment on a 14:10 h light/dark cycle (Lights on: 6:00 AM, Lights off: 8:00 PM). Animals were allowed several days to habituate to the facility prior to the beginning of the study.

### 2.2 Trigeminal ganglion cell dissociation

Trigeminal ganglion cells from 6 to 8 week old mice were acutely dissociated as described by Malin and colleagues with minor modifications (Gregus et al., 2012, Malin et al., 2007). Mice were anesthetized with 5% isoflurane and perfused transcardially with 10 ml of ice-cold sucrose-containing artificial cerebrospinal fluid (aCSF) (sucrose-aCSF) that contained (in mM): NaCl 95, KCl 1.8, KH_2_PO_4_ 1.2, CaCl_2_ 0.5, MgSO_4_ 7, NaHCO_3_ 26, glucose 15, sucrose 50 kynurenic acid 1, oxygenated with 95% O_2_, 5% CO_2_; pH 7.4. Trigeminal ganglia were quickly dissected and enzymatically digested at 37 °C for 20 min in Hanks’s balanced salt solution (HBSS; Invitrogen, Carlsbad, Ca) containing 60 units/ml papain (Worthington Biochemical, Lakewood, NJ) and 0.3 mg/ml L-cysteine (Sigma, St. Louis, MO), followed by 20 min in HBSS containing 4 mg/ml collagenase 2 (Worthington Biochemical, Lakewood, NJ) and 4.7 mg/ml dispase II (Sigma, St. Louis, MO). The ganglia were mechanically triturated by passing them 2–3 times through a 200 μl pipet tip. Myelin and nerve debris were removed by layering the cell solution over a Percoll gradient (12.5% over 28%) in L15 complete media (Thermo Fisher, Waltham, MA) containing 5% FBS (Thermo Fisher, Waltham, MA) and Penstrep (1000 U/ml, Thermo Fisher, Waltham, MA), and centrifuged at 1300 ×*g* for 10 min. The pellet was re-suspended in Ham’s F12 medium (Radnor, PA) containing 10% FBS and Penstrep. Cells were then plated coverslips precoated with 0.1 mg/ml poly-l ornithine (Sigma, St. Louis, MO) and 5 μg/ml mouse laminin (Thermo Fisher, Waltham, MA). Cells were used for calcium imaging within 18 h of dissociation.

### 2.3 Calcium imaging

TNs were incubated at room temperature in L15 media containing 10% FBS, 5 mM HEPES, 5 mM glucose and 2.5 μM Fura-2 AM (Invitrogen, Carlsbad, CA) with 0.02% pluronic f127 (Invitrogen, Carlsbad, CA) for 20 min. Individual coverslips were placed in a recording chamber superfused with artificial cerebrospinal fluid (aCSF; in mM: NaCl 127, KCl 1.8, KH_2_PO_4_ 1.2, CaCl_2_ 2.4, MgSO_4_ 1.3, NaHCO_3_ 26, glucose 15) at 5 ml/min by a solenoid-controlled six-channel Perfusion Valve Control Systems (Harvard Apparatus, Hamden, CT USA). Fluorescent images were collected using a Nikon FN1 upright microscope with a 79,000 ET FURA2 Hybrid filter set and a Photometrics CoolSNAP HQ^2^ camera. Relative intracellular Ca^2+^ levels were determined by measuring the change in ratio of fluorescence emission at 510 nm in response to excitation at 340 and 380 nm Paired images were collected at 0.67 frames/second. Cell viability was confirmed using a brief (10 s) application of 50 mM K^+^ to evoke an increase in [Ca^2+^]_i_. Only cells that demonstrated a positive response to 50 mM K^+^ (an increase in fura-2 fluorescent ratio >10% above baseline) both before and after experimental procedures were considered viable.

### 2.4 Compounds

11-hydroxy-12,13-*trans*-epoxy-(*9Z*)-octadecenoate (11H-12,13E-LA) and 11-hydroxy-9,10-*trans*-epoxy-(*12Z*)-octadecenoate (11H-9,10E-LA) and their 11-keto derivatives were prepared by total synthesis by G.S. Keyes, the details of which will be reported elsewhere. Synthesized compounds were purified with flash chromatography and/or normal-phase high-performance liquid chromatography. Nuclear magnetic resonance (NMR) analysis indicated chemical shifts and coupling constants consistent with each chemical structure. Hydroxyepoxy- or keto-epoxy-octadecenoates were analyzed by proton NMR in deuterated chloroform as their free acids or methyl esters as indicated.

Compounds in EtOH were stored at −80 °C until use. Immediately prior to use, the EtOH was dried under a stream of nitrogen and then redissolved at working concentrations in aCSF. All other compounds were purchased from Sigma (St. Louis, MO). Using this method, vehicle alone did not elicit any Ca^2+^ responses.

### 2.5 Statistics

For Ca^2+^ imaging, peak Ca^2+^ responses represent the average change of all viable cells in fluorescence ratio (Δ340/380) ± SEM. For each of the drug application protocols performed for calcium imaging, TNs were collected from a single mouse, and at least one coverslip of TNs from each mouse was used for each experiment. P values were determined by one- and two-way ANOVA using GraphPad software version 6.0 (San Diego, CA).

## 3 Results

### 3.1 Calcium signaling in response to oxylipins

Here, we sought to determine if 11-HEL and 11-KELs activate Ca^2+^ signaling in TNs. To test this hypothesis, we measured the Ca^2+^ signals of TNs in response to each compound. Fig. 2 illustrates the experimental design and Ca^2+^ signaling responses. A total of 1748 TNs were imaged for sensitivity to various oxylipins. All cells were tested with high potassium solution (50 mM) before and after 5 min exposure to test compound to insure cell viability. Cells were then tested for sensitivity to PGE_2_ (10 μM; 3 min), AITC (100 μM; 60 s) and capsaicin (1 μM; 10 s).

**Fig. 3:**
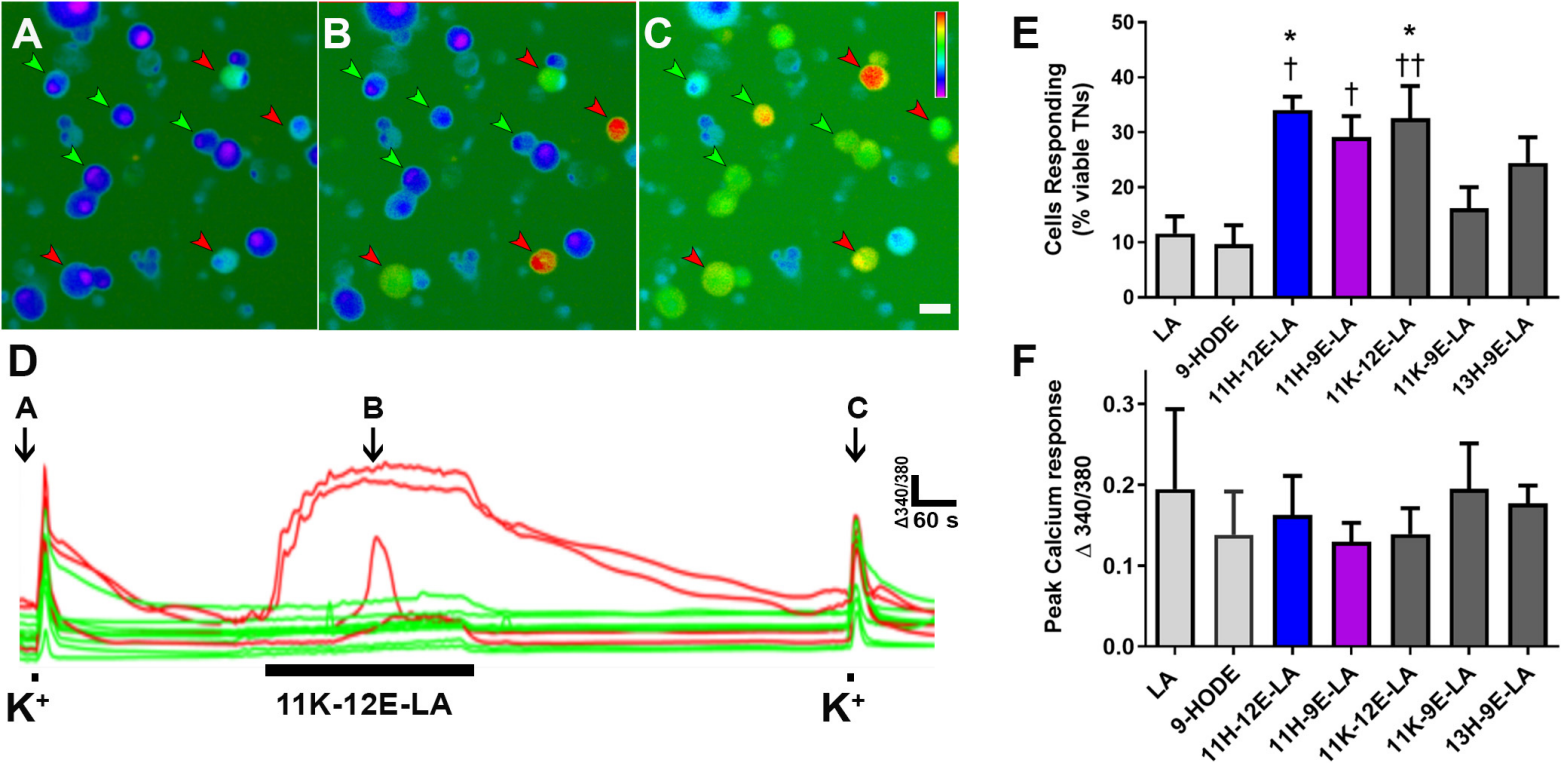
11-hydroxy-epoxide and 11-keto-epoxide derivatives of LA elicit Ca^2+^ transients in subpopulations of TNs. Representative micrographs of dissociated mouse TNs at rest (A), during exposure to 5 μM 11K-12,13E-LA (B) and 50 mM K^+^ (C). Scale bar = 10 սm (D). Representative traces showing traces of Ca^2+^ signals in response to 50 mM K^+^ (to confirm cell viability) and 11K-12,13E-LA. Arrows in (D) indicate the time point of images shown in (A-C). Green traces illustrate cells that responded only to 50 mM K^+^ (depicted by green arrowheads in A–C) while red traces illustrate cells that also responded to 5 μM 11K-12,13E-LA (depicted by red arrowheads in A–C). (E). Percent of viable (ie responsive to KCl) sensory neurons that responded to LA, 9-HODE or each 11-hydroxy-epoxide- or keto-epoxide- LA derivative (5 μM, 5 min exposure). (F) Average peak magnitude of Ca^2+^ signal is shown for each compound. Data represent mean ± SEM for 5–8 mice. *P < 0.05 compared to LA, †P < 0.05, ††P < 0.01 compared to 9-HODE.

We first compared Ca^2+^ responses to each oxylipin at 5 μM. Examples of TN responses to test compounds and high potassium solution are shown in Fig. 3A–D. 11K-12,13E-LA shown in Fig. 3A–D, produced Ca^2+^ responses in 32.5 ± 5.9% of neurons. The proportion of viable (ie responsive to KCl) neurons that responded to test compounds ranged from 16.2 ± 3.8 cells % (11K-9,10E-LA) to 34.0 ± 2.4% (11H-12,13E-LA). LA and 9-HODE (5 μM, 5 min) elicited responses in 11.6 ± 3.1% and 9.7 ± 3.4% of TNs, respectively. Fig. 3E shows that 11H-12,13E-LA, 11H-9,10E-LA and 11K-12,13E-LA produced Ca^2+^ responses in a significantly higher proportion of neurons compared to either LA or 9-HODE (F_(6, 36)_ = 5.12, P = 0.0007). While the proportion of TNs that responded to these oxylipins was greater, the average peak magnitude of Ca^2+^ responses did not differ among any of these compounds including LA and 9-HODE (Fig. 3F).

**Fig. 4:**
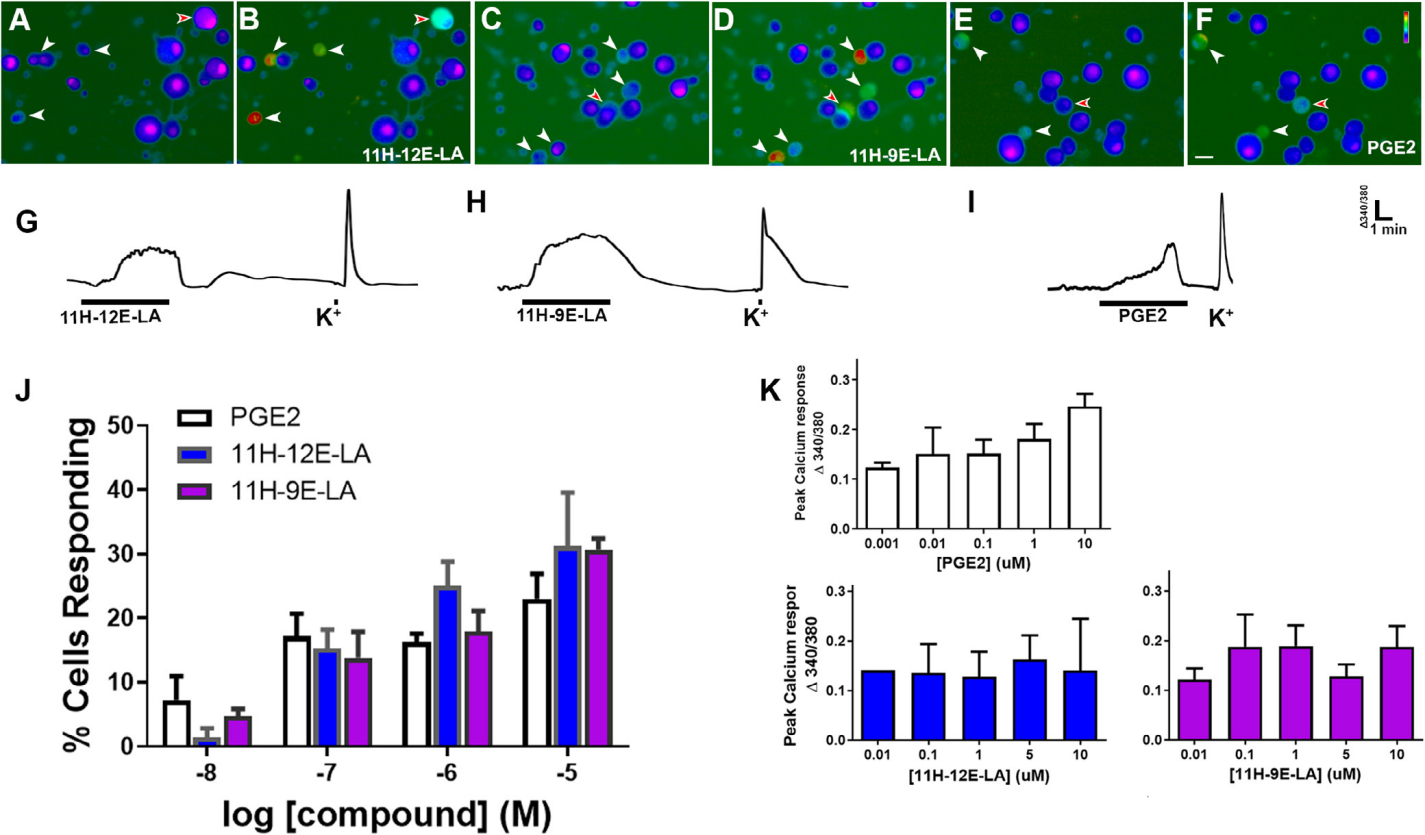
11-hydroxy-epoxide and 11-keto-epoxide derivatives of LA elicit concentration-dependent responsiveness in TNs. (A-F) Representative micrographs of dissociated mouse TNs are shown before (A, C, E) and during (B, D, F) application of 5 μM 11H-12,13E-LA, 11H-9,10E-LA, and PGE_2_, respectively. Arrowheads indicate Fura-2–loaded cells exhibiting Ca^2+^ signals in response to compound. Responses in cells depicted by red arrowhead are shown in (G-I). Representative traces showing traces of Ca^2+^ signals in response to 11H-12,13E-LA (G) 11H-9,10E-LA (H) or PGE_2_ (I) followed by 50 mM K^+^. (J) Concentration-response curves illustrate the increase in the number of cells responding to 11H-12,13E-LA, 11H-9,10E-LA and PGE_2_. (K) Average peak magnitude of Ca^2+^ signal is shown for increasing concentrations each compound. Data represent mean ± SEM for 5–8 mice. Scale bar = 20 μm.

To further characterize oxylipin-induced neuronal activation, we measured Ca^2+^ responses to increasing concentrations of the two HELs, 11H-12,13E-LA and 11H-9,10E-LA, compared to PGE_2_ (Fig. 4A–I), a well-established mediator of headache and craniofacial pain (Alstergren and Prostaglandin, 2000, Antonova et al., 2012).

We observed a concentration-dependent increase in the proportion of neurons responding to each compound. Two-way ANOVA comparing 11H-12,13E-LA, 11H-9,10E-LA and PGE_2_ revealed statistically similar responsiveness (P > 0.05). While the proportion of neurons responding increased, the magnitude of Ca^2+^ response did not vary with concentration of either compound (Fig. 4K; P > 0.05).

### 3.2 Sensitivity to oxylipins overlaps incompletely with PGE_2_ AITC and capsaicin

Table 1 shows the incidence of responsiveness to test compounds, LA and 9-HODE with responsiveness to other pain mediators. Of the 219 neurons that responded to a test compound, 40.14% also responded to PGE_2_. A substantial fraction of oxylipin-sensitive neurons also responded to AITC (58.0%) and 32.9% responded to capsaicin. While, most oxylipin-sensitive neurons responded to one or more of these three additional agents 20.6% did not respond to any other agents tested (Table 1).

**Table 1:** Incidence of responsiveness to pain mediators in oxylipin-sensitive trigeminal ganglion neurons.

	Overlapping responsiveness		
	PGE_2_ (10 μM)	AITC (100 μM)	Capsaicin (1 μM)
11H-12E-LA	13/44 **(29.5%)**	22/44 **(50.0%)**	17/44 **(38.6%)**
11H-9E-LA	20/46 **(43.5%)**	23/46 **(50.0%)**	15/46 **(32.6%)**
11K-12E-LA	14/45 **(31.1%)**	23/45 **(51.1%)**	6/45 **(13.3%)**
11K-9E-LA	13/37 **(35.1%)**	29/37 **(78.4%)**	9/37 **(24.3%)**
13H-9E-LA	28/47 **(59.5%)**	30/47 **(63.8%)**	25/47 **(53.2%)**

total	**88/219 (40.2%)**	**127/219 (58.0%)**	**72/219 (32.9%)**

LA	**13/25 (52.0%)**	**19/25 (76.0%)**	**11/25 (40.0%)**
9-HODE	**8/16 (50.0%)**	**10/16 (62.5%)**	**8/16 (50.0%)**

## 4 Discussion

LA is an abundant polyunsaturated fatty acid in many peripheral tissues, where it can act as a substrate for enzymatic and free-radical mediated peroxidation, with subsequent conversion into numerous well-known LA derived oxylipins, including hydroxy-octadecadienoic acids (HODEs), epoxy-octadecenoic (EpOMEs) and dihydroxy-octadecenoic acids (DiHOMEs) (Hargreaves and Ruparel, 2016, Ramsden et al., 2012, Vangaveti et al., 2016, Lundstrom et al., 2012, Ramsden et al., 2016). In preclinical models, HODEs, EpOMEs and DiHOMEs have been observed to participate in numerous (patho)physiological processes during inflammatory pain including mechanical and thermal hyperalgesia (Zimmer et al., 2018, Patwardhan et al., 2010, Patwardhan et al., 2009). We recently identified two 11-hydroxy-epoxide- derivatives of LA—11H-12,13E-LA and 11H-9,10E-LA—that are present in inflamed human skin and were observed (Ramsden et al., 2016) to sensitize rat dorsal root ganglia neurons to release CGRP, a mediator that plays a key role in migraine pathogenesis (Iyengar et al., 2019, Ramsden et al., 2017). Moreover, in a small randomized trial in patients with chronic headaches, diet-induced reductions in 11H-12,13E-LA in plasma correlated with pain relief, further suggesting that these two LA–derived mediators might contribute to sensory signaling (Ramsden et al., 2017).

Here we expand upon these initial findings by describing a family of linoleic acid-derived oxylipins that activate TNs, suggesting their potential role in trigeminally-mediated pain including headache and facial pain. We demonstrate two 11-hydroxy-epoxides increased proportions of responsive TNs in a concentration-dependent fashion, similar to PGE_2_. Further investigation revealed that exposure produced Ca^2+^ responses with high potency, at μM range, comparable to well-known pain mediators, LA and 9-HODE (Patwardhan et al., 2010, Patwardhan et al., 2009). Together, high potency and concentration-dependent responses might suggest a direct effect on a receptor such as TRPV1 as is the case in the context of other well-characterized linoleic acid derivatives (Patwardhan et al., 2009). If this were true for the compounds we tested, we would expect a complete overlap between cells that respond to that oxylipin and the TRPV1 agonist, capsaicin. However, we observed an incomplete (13.3–53.2%) overlap between response to oxylipin and capsaicin. Overlap between oxylipins and either PGE_2_ or AITC was also incomplete, suggesting an as of yet unidentified mechanism independent or in addition to direct activation of TRPV1, TRPA1 or PGE_2_ receptors. Previous studies demonstrate that lipids, including 9-HODE may sensitize TRP channels (Patwardhan et al., 2010). Likewise, they can cause enhanced activity of sensory neurons by mechanisms involving activation of G-protein coupled receptors (GPCRs) and/or modulating the activity of ion channels in peripheral sensory neurons (Lim and Kim, 2016, Moran et al., 2011, Pinho-Ribeiro et al., 2017, Schafer et al., 2020, Sisignano and Bennett, 2014). Such activation mediates second messenger signaling cascades that sensitize ion channels, including TRPV1 and TRPA1, leading to increased activity of peripheral sensory neurons. Recently, Schafer and colleagues demonstrated that the omega-3 lipid 17,18-EEQ sensitizes TRPV1 and TRPA1 in sensory neurons through a G_s_-coupled prostacyclin receptor and subsequent PKA activation (Schafer et al., 2020). Alternatively, these lipids may exert effects via direct binding and gating of TRPV1/TRPA1 multimeric complexes, binding to TRPV1 and transactivation of TRPA1, or binding to a G protein-coupled receptor and signaling via second messenger systems to increase membrane trafficking of TRPV1/TRPA1. Recent studies demonstrate functional interaction of these receptors in sensory neurons including TNs (Caterina et al., 2000, Salas et al., 2009).

The two 11-HELs share a 3-hydroxy-Z-pentenyl-E-epoxide moiety with each other and with previously described bioactive hepoxilin B3 derivatives of arachidonic acid (Anton and Puig, 1998, Anton et al., 2002, Pace-Asciak, 2015). Since hepoxilins have been implicated in algesic and inflammatory responses (Gregus et al., 2012), it is possible that this substructure could mediate nociceptor sensitization. Notably, however, one of the two 11-KELs tested (11K-12,13E-LA), activated TNs in a similar manner as the two 11-HELs, indicating that this putative pharmacophore is not the sole determinant of their activity in this model. Future studies comparing the activities of hepoxilins, 11-HELs, 11-KELs and related compounds derived from other fatty acid backbones are needed to better understand the structure-function relationships of these lipid families. Interestingly, these oxylipins show similar profiles as LA and 9-HODE, with greatest responsiveness to AITC and intermediate responsiveness to capsaicin and PGE_2_. Thus, it seems plausible that these oxylipins play a similar role to well-established pain mediators. Of note, three compounds (11H-12,13E-LA, 11H-9,10E-LA and 11K-12,13E-LA) produced Ca^2+^ responses in significantly more TNs than LA or 9-HODE, suggesting they may be potent activators of nociceptive signaling. While more studies are needed to characterize the mechanism of activation, these findings support the concept that 11-HEL and 11-KELs could augment trigeminal nociceptive responses and contribute to headaches and other craniofacial pain syndromes.

This study has several important limitations. 11-HEL and 11-KEL compounds were synthesized as racemic mixtures. Future studies are therefore needed to attribute biological actions to an individual stereoisomer. The 11-KELs are highly reactive and exhibit limited solubility, making accurate preparations challenging. Therefore, concentration-dependent effects should be interpreted with some caution. Of note, some endogenous bioactive lipid families like the endocannabinoids, act in an entourage fashion such that receptor signaling is enhanced when they occur in combination as a result of either competition for synthesis or turnover, or by directly enhancing receptor activation (Ben-Shabat et al., 1998, Smart et al., 2002). The temporal profiles and concentration may also differ based on the particular pain state. Therefore, future analytical studies are needed to identify and quantify concentrations of these compounds in TN-innervated tissues.

It should also be noted that our studies were conducted in TNs from un-injured mice and therefore do not reflect responses during a hyper-excitable state. Peripheral sensory neuron sensitization has been well established in response to inflammatory mediators and low pH (Sessle, 2011, Hucho and Levine, 2007, Chichorro et al., 2017). These oxylipins potentially participate in additional cellular responses in pathological conditions. Although pre-exposure to 110 mM K^+^ for 15 min in smooth muscle preparations has been shown to activate signaling systems such as RhoA kinase (Ratz et al., 2009), we have not determined if our brief pre-exposure to 50 mM K^+^ alters TN sensitivity. Future studies are needed to identify the specific substructures mediating nociceptor sensitization, and to characterize the signaling pathways responsible for the observed effects. Animal behavior studies (i.e. meningeal infusions) are needed to clarify whether these lipids impact headache and craniofacial pain, and to place their roles into context of established pain mediators. Importantly, given that sex is a biological determinant of pain (Fillingim et al., 2009), male and female animal models or human participants should be included in future studies.

## 5 Conclusions and future directions

Collective findings demonstrate that 11-HEL and 11-KEL increase TN excitability. Future studies are needed to clarify signaling pathways and to determine if a 11-HEL or 11-KEL contribute to headache or facial pain.

## Declaration of Competing Interest

The authors declare that they have no known competing financial interests or personal relationships that could have appeared to influence the work reported in this paper.
